# Laurence-Moon-Bardet-Biedl Syndrome: A Case Report

**DOI:** 10.7759/cureus.5618

**Published:** 2019-09-10

**Authors:** Bilal Ahmed Khan, Ashar Shahid, Maaz Bin Nazir, Kiran Shafiq Khan, Avinash Punshi

**Affiliations:** 1 Dow Medical College, Dow University of Health Sciences (DUHS), Karachi, PAK; 2 Internal Medicine, Dow University of Health Sciences (DUHS), Karachi, PAK; 3 Internal Medicine, Civil Hospital Karachi, Karachi, PAK

**Keywords:** polydactyly, bardet biedl, laurence moon syndrome, obesity, hypogonadism, retinitis pigmentosa

## Abstract

Laurence-Moon-Bardet-Biedl syndrome (LMBBS), a rare autosomal recessive defect, mostly occurs in children born from consanguineous marriages. The major features of this syndrome are cone-rod dystrophy, polydactyly, obesity, learning disabilities, hypogonadism in males, renal anomalies, nystagmus, speech disorders, developmental delay, polyuria/polydipsia, ataxia, and poor coordination/clumsiness. In this report, we present a case of a 19-year-old man with pain and swelling of the left ankle and knee joints because of which he could not walk, with an onset of loose stools since a week. He presented with multiple non-itchy hyperpigmented macules on his face and back, polydactyly in his left foot, central obesity, proteinuria, macrocytic anemia, low intelligence quotient, reduced power in the left lower limb, reduced plantar reflexes, nystagmus, pigmented black lesions in the temporal retina on fundoscopy, a micropenis, absent pubic and axillary hair, and a small scrotum containing testes. The patient was diagnosed with LMBBS.

## Introduction

Laurence-Moon-Bardet-Biedl syndrome (LMBBS) is a rare ciliopathic, pleiotropic autosomal recessive defect that mostly occurs in children born from consanguineous marriages. These patients generally show symptoms within the first ten years of life, with poor night vision being the first [1]. Incidence rates in North America and Europe vary from 1:140,000 to 1:160,000 live births. Conversely, in Kuwait and Newfoundland, the rate is much greater, with an estimated frequency of 1:13,500 and 1:17,500, respectively [2]. The presence of four primary features on their own or three primary coupled with two secondary features are the clinical grounds for making a diagnosis. Cone-rod dystrophy, polydactyly, obesity, learning disabilities, hypogonadism in males, and renal anomalies are classified as primary features, whereas secondary features include speech disorders, brachydactyly, developmental delay, polyuria/polydipsia, ataxia, poor coordination/clumsiness, diabetes mellitus, left ventricular hypertrophy, hepatic fibrosis, spasticity, and hearing loss. Apart from these features, short stature, crowding of teeth, hypermobile or lax joints, and early osteoarthritis are also reported [3].

Moreover, nystagmus and constricted peripheral visual fields are commonly noted. Constricted arterioles, waxy disc pallor, and peripheral pigmentation changes, such as pigment atrophy and bone specular pigmentation plus areas of white deposits, are the major changes observed in the fundus. At an early stage, degenerative changes are observed in the maculae of patients, with a persistent decrease in their central vision, thus making them legally blind by the age of 30 years. Diffuse photoreceptor disease is confirmed by electroretinography, thus differentiating LMBBS from retinitis pigmentosa, which is not associated with any systemic disease [4].

More than half of the total cases reveal that women are more commonly affected than men. Moreover, functional and morphological abnormalities are observed in up to 90% of the affected patients. The renal abnormalities occur with a range of activities, often causing substantial morbidity, and the autopsy records reveal it to be the chief cause of mortality [5].

## Case presentation

A 19-year-old man, with hepatitis C for four years, asthma since childhood, and LMBBS diagnosed four years ago, presented in the medical outpatient department of Dr. Ruth Pfau Civil Hospital Karachi in 2018, with the complaint of pain and swelling in the left ankle and both the knee joints, and loose stools from one week. According to the patient, he was in his usual state of health two weeks before, after which he experienced multiple episodes of foul-smelling, whitish-yellow, watery stools every day. The loose stools were non-bloody and not associated with vomiting, nausea, or fever. This problem settled after a week, at which time the patient complained of being unable to walk because of painful swelling in the left ankle and knees, although there was no history of a fall or trauma.

His mother stated that he was mentally unstable since childhood, as he showed delayed physical and mental growth. She specifically mentioned that he learned to walk and speak at the age of approximately two and a half years and four years, respectively. Moreover, the child was born of a consanguineous marriage. The mother had never smoked and did not take any medication during the pregnancy. He was born through normal vaginal delivery, without any history of birth asphyxia, feeding difficulty, or cyanosis, and was immunized periodically. His brother, conceived from the same parents, had no such complications.

**Figure 1:**
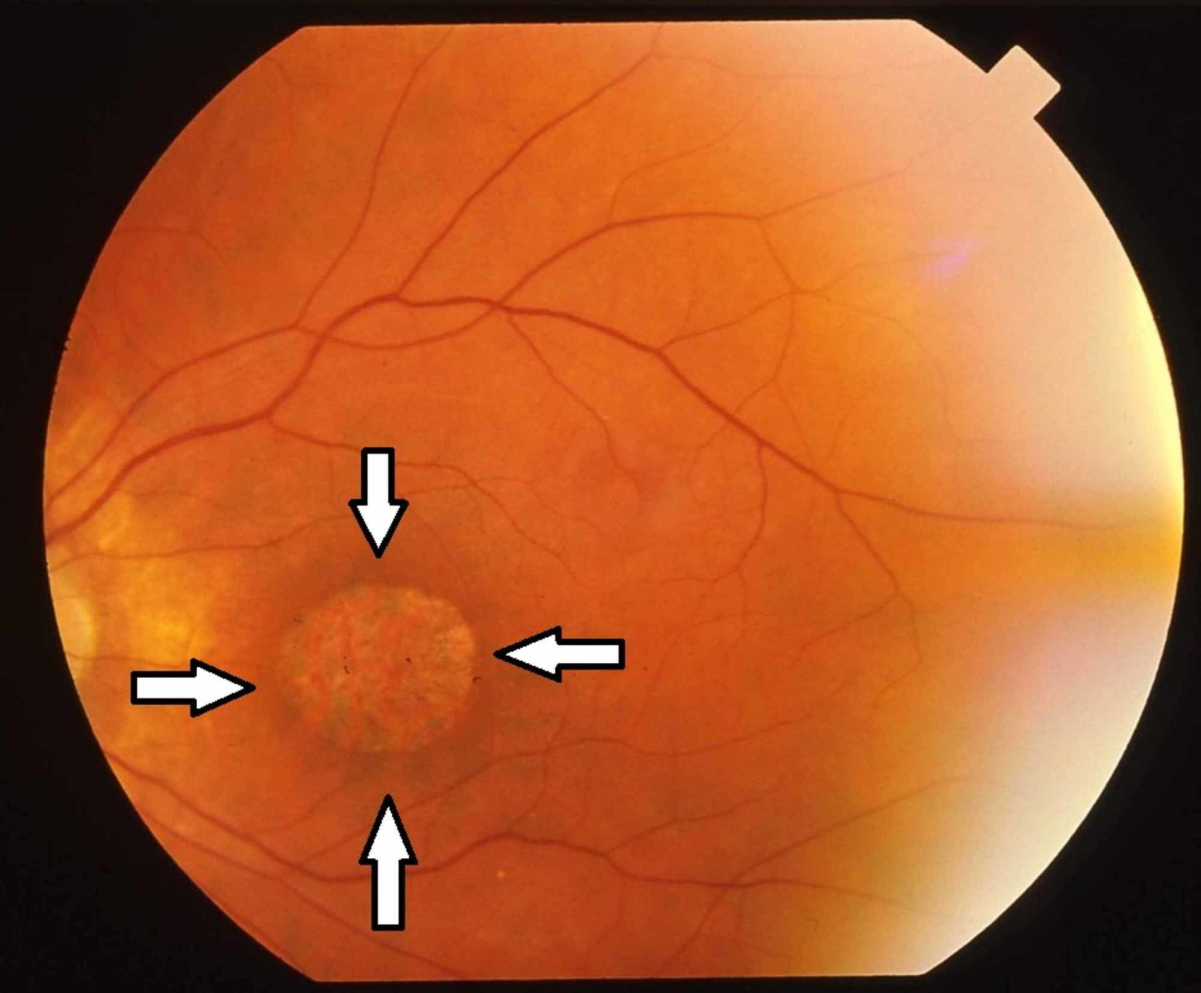
Retinitis Pigmentosa

The clinical examination showed a young man of average height, having central obesity, and who was well oriented with time, place, and person, with frequent eye blinking and head nodding. Multiple non-itchy hyperpigmented macules were observed on the face and back. Polydactyly was seen in the left foot. The central nervous system examination revealed low intellectual quotient, a 13/15 Glasgow Coma Score, normal higher mental functions, and an intact gag and cranial reflex. The patient was unable to button his clothes, which is a marker of a cerebral defect. The muscle bulk and tone were normal in all limbs with regard to the power and reflexes, and the left lower limb showed a power grading of 4/5, whereas the plantar reflexes of both legs were reduced. An eye examination revealed nystagmus in both eyes, and the subsequent fundoscopy showed pigmented black lesions in the temporal retina, i.e., retinitis pigmentosa (Figure 1). The genital examination revealed a micropenis of approximately 1.7 cm; smaller than regular scrotum of approximately 2 cm, with intact testes; and lack of pubic and axillary hair. All vitals were normal. The urine DR showed proteinuria and blood tests showed that anti-HCV antibodies were positive. The complete blood count results revealed a white blood cell count of 13.9 × 103/L with hemoglobin of 9.4 g/dL, a mean corpuscular volume of 102 fL, a mean corpuscular hemoglobin level of 33.2 pg/cell, and a platelet count of 210 × 103/L. The red blood cell morphology was normochromic, anisocytosis, and macrocytosis. He was also obese with a body mass index of 31 kg/m2.

The patient had a history of multiple hospital admissions: he had been admitted four years ago for bloody diarrhea, one year ago for severe vomiting, and one week before for loose stools. He was suspected of having celiac disease; however, laboratory reports including antitransglutaminase, immunoglobulin G, and immunoglobulin A all were within normal ranges. In addition, the antirheumatic factor was negative. Laboratory tests showed serum antineutrophil cytoplasmic antibodies and antinuclear antibody were negative; therefore, vasculitis was ruled out.

There is no definitive treatment for this condition, although early diagnosis and symptomatic, supportive, and rehabilitative measures can reduce disability. The patient’s mother was advised to strictly follow the given dietary modification plan and the prescribed oral hypoglycemic drugs, testosterone, vitamin A supplements etc.

The mother had provided informed consent for publishing this case report; however, she refused to give permission for taking photos of her child. To protect the identity of the patient, we have not stated facts that may reveal his identity and all data have been published anonymously.

## Discussion

The rationale behind this study is the scarcity of available information about LMBBS in Pakistan. Our patient being male, whereas most reported cases were females, further adds to the fact.

Confusion still exists between Lawrence-Moon syndrome (LMS) and Bardet-Biedl syndrome (BBS). Pigmentary retinal degeneration, mental retardation, and hypogonadism are common in both, whereas spastic paraplegia is predominant in LMS and polydactyly and obesity are seen in BBS. Because of some common features, some researchers believe BBS to be a part of LMS [6]. BBS is a rare ciliopathic disorder that affects 12 genes (BBS1 to BBS12) which form proteins essential in the functioning of the cilia, thereby causing structural and functional anomalies [7]. Two defective genes, one from each carrier parent, yield an offspring affected by LMBBS; thus, the probability, in this case, was 25%. Therefore, genetic counseling is beneficial for both patients and their families [8]. The modified diagnostic criteria are given in Table 1.

 

**Table 1:** Modified diagnostic criteria for Bardet-Biedl syndrome

Primary Features	Secondary Features
Rod-cone dystrophy, Polydactyly, Obesity, Learning disabilities, Hypogonadism in males, Renal anomalies.	Speech disorder/delay, Strabismus/cataracts/astigmatism, Brachydactyly/Syndactyly, Developmental delay, Polyuria/Polydipsia(Nephrogenic Diabetes Insipidus), Ataxia/poor coordination/imbalance, Mild spasticity, Diabetes mellitus, Dental crowding/Hypodontia/Small roots/High arched palate, Left ventricular hypertrophy/Congenital Heart disease, Hepatic fibrosis.

Consanguinity is commonly practiced in various Middle Eastern countries, such as Kuwait, Saudi Arabia, Iran, and Pakistan. It has been a chief contributing factor to disease frequency. Families in these countries have approximately 0.1% homozygous genes of the total genome. In Pakistan, more than half of all marriages are consanguineous in nature, with 80% of them among first cousins, thereby increasing the possibility of homozygous mutations. Despite this disturbing fact, the frequency of LMBBS is still unknown in Pakistan, and the majority of cases go undiagnosed. Until date, only nine cases with these mutations have been reported [9].

Our patient being a known asthmatic is also significant, as it is a condition thought to be associated with LMBBS but not entirely part of the major diagnostic criteria [10]. The renal abnormalities in LMBBS patients are as frequent as the pentad of cardinal features of this disease. As there is a significant risk of progression to end-stage renal failure, particularly in the third and fourth decades of life, blood pressure control and routine tests must be particularly emphasized [11].

The treatments available for LMBBS are mainly toward managing the manifestations of the illness. Physical therapy aimed toward improving strength helps. Exercise can reduce the symptoms of spasticity. A dedicated regimen of nutritious, well-balanced meals and regular exercise is recommended, as there is an increased incidence of diabetes and abnormal cholesterol levels in patients with LMS. A low protein diet also slows the progression of renal diseases in BBS [12]. The poor functional capacity of the anterior pituitary gland, resulting in slow metabolism, poor growth, and impaired fertility, can be managed with hormone replacement therapies. Levothyroxine can aid in increasing the body metabolism, resulting in reduced lethargy, hair loss, and obesity. Growth hormone supplementation reduces the psychosocial burden of short stature, whereas testosterone supplementation can be given in patients with markedly low levels to prevent underdeveloped genitalia. Accessory digits are generally nonfunctional and can be removed for cosmetic purposes. Typically, retinal dystrophy is the first symptom that arises before the age of 10 years but affects almost all patients below the age of 20 years [13]. Glasses can be used to treat this, and regular ophthalmologist visits are recommended [14].

However, despite exhibiting the manifestations of LMBBS from an early age, our patient was not diagnosed until he was 16 years, by which time a significant amount of damage had already been done. Ideally, during puberty, doctors should refer these patients to an experienced counselor, as it is an extremely stressful time for them, which unfortunately our patient missed.

## Conclusions

Considerable morbidity and mortality are imposed by LMBBS on children. An earlier diagnosis can help professionals more effectively recognize and therefore manage this condition. Because of its rarity and the involvement of different systems, it is generally missed even by specialists, as observed in this case. A timely and thorough management plan would allow these children to integrate better into society and thrive fully. Furthermore, both parents should undergo genetic counseling, especially those with a history of consanguineous marriages in the family. Marriages outside of the family should also be promoted. 
